# Prevalence and characterization of *Cryptosporidium* in sika deer from Northern China

**DOI:** 10.1051/parasite/2025029

**Published:** 2025-06-11

**Authors:** Yan Tang, Nian-Yu Xue, Yang Gao, Zhen-Qiu Gao, Hong-Di Zhuang, Guang-Rong Bao, Hong-Wei Cao, Jing Liu, Jian-Ming Li, Shuo Liu

**Affiliations:** 1 College of Life Sciences, Changchun Sci-Tech University Shuangyang Jilin Province PR China; 2 College of Pharmacy, Guizhou University of Traditional Chinese Medicine Guiyang Guizhou Province PR China; 3 College of Veterinary Medicine, Yangzhou University Yangzhou Jiangsu Province PR China; 4 State Key Laboratory for Animal Disease Control and Prevention, Harbin Veterinary Research Institute, Chinese Academy of Agricultural Sciences (CAAS) Harbin Heilongjiang Province PR China; 5 School of Pharmacy, Yancheng Teachers University Yancheng Jiangsu Province PR China; 6 College of Animal Science and Technology, Jilin Agricultural University Changchun Jilin Province PR China; 7 College of Veterinary Medicine, Qingdao Agricultural University Qingdao Shandong Province PR China

**Keywords:** *Cryptosporidium* sp., Sika deer, Prevalence, Subtype, China

## Abstract

*Cryptosporidium* spp. are important zoonotic parasites that can cause moderate to severe diarrhea in humans and animals. However, the epidemiological data of *Cryptosporidium* in sika deer in China need to be updated. In this study, a total of 466 fecal samples were collected from sika deer in Shandong, Jilin, Liaoning, and Heilongjiang provinces. Nested PCR was used to amplify the *SSU* rRNA gene to detect *Cryptosporidium* spp. The results showed that the overall infection rate of *Cryptosporidium* spp. was 14.81%, with no significant differences among regions (*p* = 0.05). The highest infection rate was found in Heilongjiang Province (23.60%) and the lowest in Jilin Province (10.71%). The infection rate in summer (23.61%) seemed higher than that in autumn (13.20%), but the difference was not statistically significant (*p* = 0.30). Notably, young sika deer showed a significantly higher infection rate (28.21%) compared to adults (10.32%) (*p* < 0.0001). Sequence analysis identified two *Cryptosporidium* species/genotypes: *Cryptosporidium* deer genotype (98.55%) and *Cryptosporidium ubiquitum* (1.45%). Subtyping revealed that the *C. ubiquitum* isolate belonged to the zoonotic XIIa subtype. These findings provide new insights into the prevalence and genetic diversity of *Cryptosporidium* in sika deer and suggest that sika deer may act as a potential reservoir for zoonotic *Cryptosporidium* transmission.

## Abstract (fr)

Les *Cryptosporidium* spp. sont d’importants parasites zoonotiques pouvant provoquer des diarrhées modérées à sévères chez l’homme et l’animal. Cependant, les données épidémiologiques concernant *Cryptosporidium* chez le cerf sika en Chine doivent être mises à jour. Dans cette étude, 466 échantillons fécaux ont été prélevés sur des cerfs sika dans les provinces du Shandong, du Jilin, du Liaoning et du Heilongjiang. La PCR nichée a été utilisée pour amplifier le gène de l’ARNr SSU afin de détecter les *Cryptosporidium* spp. Les résultats ont montré que le taux d’infection global par *Cryptosporidium* spp. était de 14,81 %, sans différence significative entre les régions (*p* = 0,05). Le taux d’infection le plus élevé a été observé dans la province du Heilongjiang (23,60 %) et le plus faible dans la province du Jilin (10,71 %). Le taux d’infection en été (23,61 %) semblait plus élevé qu’en automne (13,20 %), mais la différence n’était pas statistiquement significative (*p* = 0,30). De manière notable, les jeunes cerfs sika ont montré un taux d’infection significativement plus élevé (28,21 %) que les adultes (10,32 %) (*p* < 0,0001). L’analyse de séquence a identifié deux espèces/génotypes de *Cryptosporidium* : *Cryptosporidium* génotype « deer » (98,55 %) et *Cryptosporidium ubiquitum* (1,45 %). Le sous-typage a révélé que l’isolat de *C. ubiquitum* appartenait au sous-type zoonotique XIIa. Ces résultats apportent de nouvelles perspectives sur la prévalence et la diversité génétique de *Cryptosporidium* chez le cerf sika et suggèrent que le cerf sika pourrait agir comme un réservoir potentiel pour la transmission zoonotique de *Cryptosporidium*.

## Introduction

*Cryptosporidium* sp. is an important zoonotic parasite that can infect a wide range of vertebrate hosts [37, 43]. It is the second leading cause of diarrhea in children, following rotavirus [7]. *Cryptosporidium* oocysts excreted by infected hosts can contaminate water and food sources, resulting in infections in both humans and animals [14, 21]. Clinical manifestations of *Cryptosporidium* infections in humans and animals commonly include diarrhea, growth retardation, and weight loss [35].

To date, more than 47 species and approximately 120 genotypes of *Cryptosporidium* have been identified using molecular methods and morphological data [13, 16, 41]. Approximately 17 species or genotypes of *Cryptosporidium* have been identified in deer. These include *Cryptosporidium andersoni*, *C. bovis*, *C. canis*, *C. hominis*, *C. meleagridis*, *C. muris*, *C. parvum*, *C. ryanae*, *C. scrofarum*, *C. suis*, *C. ubiquitum*, *C. xiaoi*, and several genotypes: *Cryptosporidium caribou genotype*, *C.* deer genotype, *C. parvum* genotype II, and *C. suis*-like genotype [10, 17, 19, 23, 26, 44].

The sika deer (*Cervus nippon*) is a small species within the *Cervinae* subfamily, valued for its significant ecological and economic importance. It is classified as a first-grade state-protected animal in China, with its primary habitat in Northeast China [28]. However, research on *Cryptosporidium* infections in sika deer remains limited. This study examined the prevalence and distribution of *Cryptosporidium* species and genotypes in sika deer from Northern China.

## Materials and methods

### Ethics approval

All procedures used in this study were approved by the Research Ethics Committee for the Care and Use of Laboratory Animals in Qingdao Agricultural University, China.

### Collection of samples

**Figure 1:**
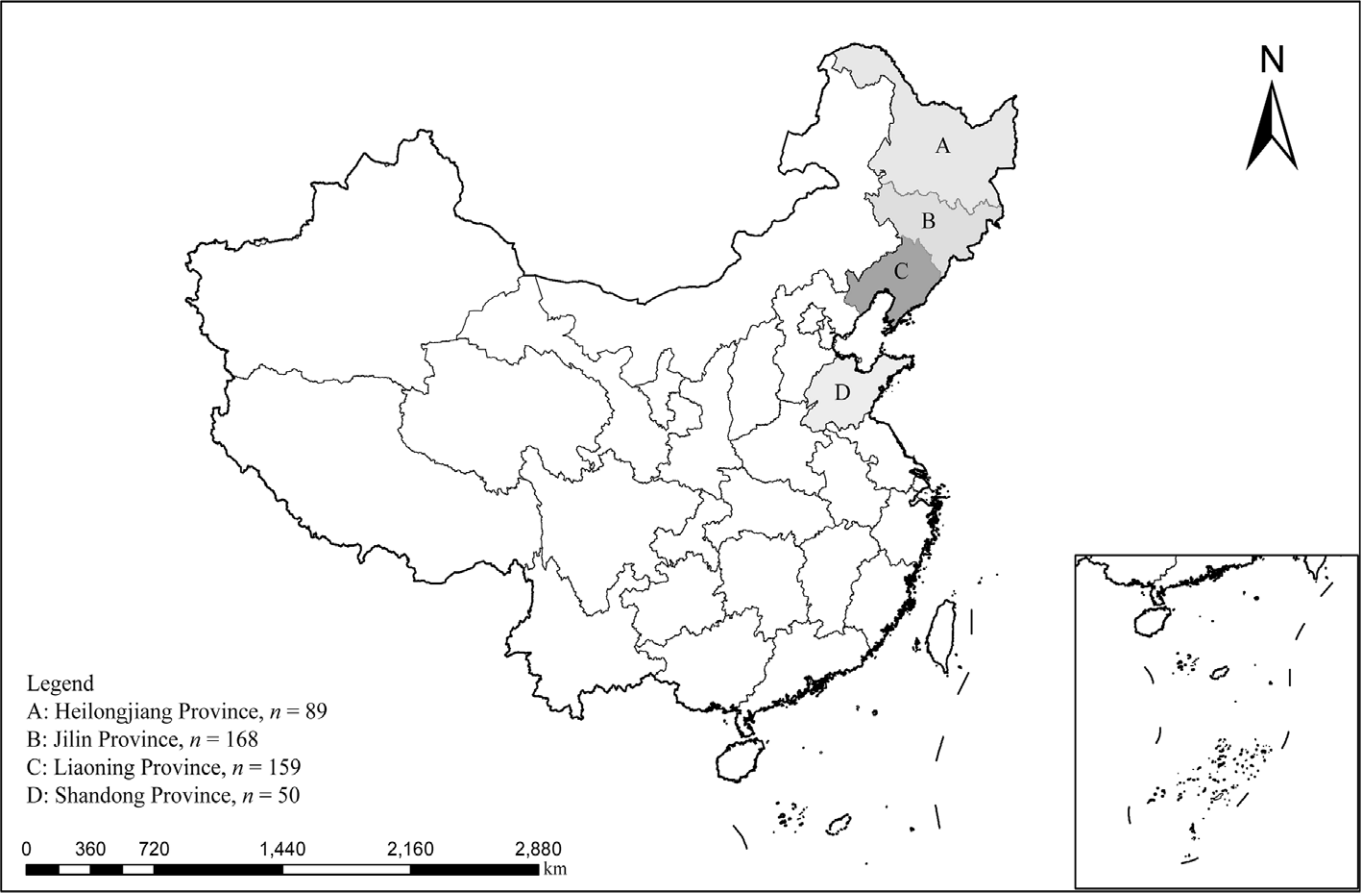
A map of the People’s Republic of China showing the sampling regions marked with letters.

A total of 466 fecal samples of sika deer were collected using random sampling from May to October 2024. Specifically, Shandong (*n* = 50), Jilin (*n* = 168), Liaoning (*n* = 159), and Heilongjiang (*n* = 89) (Fig. 1). Specific sampling methods used were as follows: immediately after defecation, fresh fecal samples were collected using sterile polyethylene gloves. To avoid contamination from the environment, only the portions of feces that had not been in contact with the ground were carefully collected. Sample source information was recorded during sampling, sika deer younger than 1.5 years old were classified as young, and those older than 1.5 years old were classified as adult. Samples were stored in frozen sampling boxes and transported to the laboratory, where they were stored at −80 °C until DNA extraction.

### Extraction of DNA and PCR amplification

Each sample was weighed to 0.5 g, and 0.2 g of glass beads were added for preprocessing. DNA extraction was then performed according to the manufacturer’s instructions. The primers used for nested PCR to amplify *SSU* rRNA genes for the identification of *Cryptosporidium* sp. were as previously described (Table 1) [32]. The first round PCR reaction system was 30 μL, containing 15 μL of 2× Mix Master Taq, 1 μL each of forward and reverse primers, 2 μL of DNA template, and 11 μL of deionized water. The reaction program was as follows: after predenaturation for 5 min at 94 °C, 35 cycles were performed, each consisting of 30 s at 94 °C, 30 s at 58 °C, 30 s at 72 °C, and a final extension for 10 min at 72 °C. The second round of PCR was performed using the same reaction system and under the same reaction procedure. The electrophoresis of 6 μL PCR products was performed on 1.0% agarose gel and observed using a UV light.

**Table 1:** Primer sequences targeting the *SSU* rRNA gene of *Cryptosporidium* sp.

Primer sequences (5′–3′)	Target fragment size (bp)	Annealing temperature (°C)
F1: GACATATCATTCAAGTTTCTGACC	763	58
R1: CTGAAGGAGTAAGGAACAACC		
F2: CCTATCAGCTTTAGACGGTAGG	587	58
R2: TCTAAGAATTTCACCTCTGACTG		
*gp60*F1: TTTACCCACACCATCTGTAGCGTCG	1044	58
*gp60*R1: ACGGACGGAATGATGTATCTGA		
*gp60*F2: ATAGGTGATAATTAGTCAGTCTTTAAT	948	55
*gp60*R2: TCCAAAAGCGGCTGAGTCAGCATC		

### Subtype identification

In order to subtype C. *ubiquitum*, a nested PCR was used to amplify a 948 bp fragment of the 60 kDa glycoprotein (*gp60*) gene (Table 1) [25]. The reaction system for both rounds of PCR was 50 μL, which include: 25 μL of 2× Mix Master Taq, 1 μL of forward primer, 1 μL of reverse primer, 19 μL of deionized water, and 4 μL DNA template. The PCR amplification procedure was as follow: pre-denaturation for 5 min at 94 °C; then 35 cycles, each cycle consisting of denaturation for 45 s at 94 °C, annealing for 45 s at 58 °C (for primary PCR) or 55 °C (for secondary PCR), and extension for 1 min at 72 °C; finally, extension for 7 min at 72 °C.

### Sequencing and phylogenetic analysis

The positive secondary PCR products were delivered to Qingdao Weilai Biotechnology Co., Ltd, China for Sanger sequencing. Sequences obtained were compared with the GenBank sequence to determine *Cryptosporidium* species. The neighbor-joining (NJ) method was used to build a phylogenetic tree and assess the relationship between obtained sequences and sequences of other *Cryptosporidium* species/genotypes, with 1,000 bootstrapping replicates to confirm data reliability. The representative gene sequences and the *gp60* gene sequence were submitted to the GenBank database under the accession numbers PV163072–PV163076 and PV642472, respectively.

### Statistical analysis

A chi-squared test in SPSS software (v.27.0, IBM Corp., Armonk, NY, USA) was used to compare the prevalence of *Cryptosporidium* in different regions. A value of *p* < 0.05 was considered statistically significant. Additionally, odds ratios (OR) and 95% confidence intervals (95% CI) were calculated using SPSS software to examine the strength of the association between *Cryptosporidium* infection and location of sika deer.

## Results

### Prevalence of *Cryptosporidium* sp.

In this study, 466 fecal samples of sika deer were tested, and 69 were positive for *Cryptosporidium*, with an overall infection rate of 14.81%. The infection rates in different provinces ranged from 10.71% to 23.60%, and there was no significant difference (χ^2^ = 7.82, *df* = 3, *p* = 0.05). The infection rate was highest in Heilongjiang province (23.6%, 95% CI 15.29–33.03), followed by Shandong province (18.00%, 95% CI 8.41–30.02) and the lowest in Jilin province (10.71%, 95% CI 6.44–15.88). The seasonal analysis showed that there was no significant difference between seasons (χ^2^ = 4.70, *df* = 1, *p* = 0.30), and the prevalence of *Cryptosporidium* in summer (23.61%, 95% CI 14.44–34.18) was higher than that in autumn (13.20%, 95% CI 10.02–16.73). Among different age groups, the prevalence of sika deer was significantly different (χ^2^ = 19.11, *df* = 1, *p* < 0.0001), and the infection rate of young sika deer (28.21%, 95% CI 20.38–36.74) was significantly higher than that of adult sika deer (10.32%, 95% CI 7.33–13.74) (Table 2).

**Table 2:** Prevalence and distribution of *Cryptosporidium* sp. in sika deer.

Factors	Category	No. tested	No. positive	% (95% CI)	Heterogeneity	OR(95% CI)
					χ^2^/df/*I*^*2*^(%)/*P*	
Region	Jilin	168	18	10.71 (6.44–15.88)	7.82/3/61.6/0.05	–
	Heilongjiang	89	21	23.60 (15.29–33.03)		
	Liaoning	159	21	13.21 (8.35–18.95)		
	Shandong	50	9	18.00 (8.41–30.02)		
Season	Autumn	394	52	13.20 (10.02–16.73)	4.70/1/78.7/0.30	–
	Summer	72	17	23.61 (14.44–34.18)		
Age	Adult	349	36	10.32 (7.33–13.74)	19.11/1/94.8/< 0.0001	Reference
	Young	117	33	28.21 (20.38–36.74)		3.42 (2.01–5.80)
Total	–	466	69	14.81 (11.71–18.36)	–	–

### *Cryptosporidium* species/genotypes and subtypes

In this study, based on *SSU* rRNA analysis, 69 *Cryptosporidium* sp.-positive samples were identified, including *C.* deer genotype (*n* = 68) and *C. ubiquitum* (*n* = 1). Additionally, the *C. ubiquitum* isolate was further subtyped using the *gp60* gene and identified as subtype XIIa.

**Figure 2:**
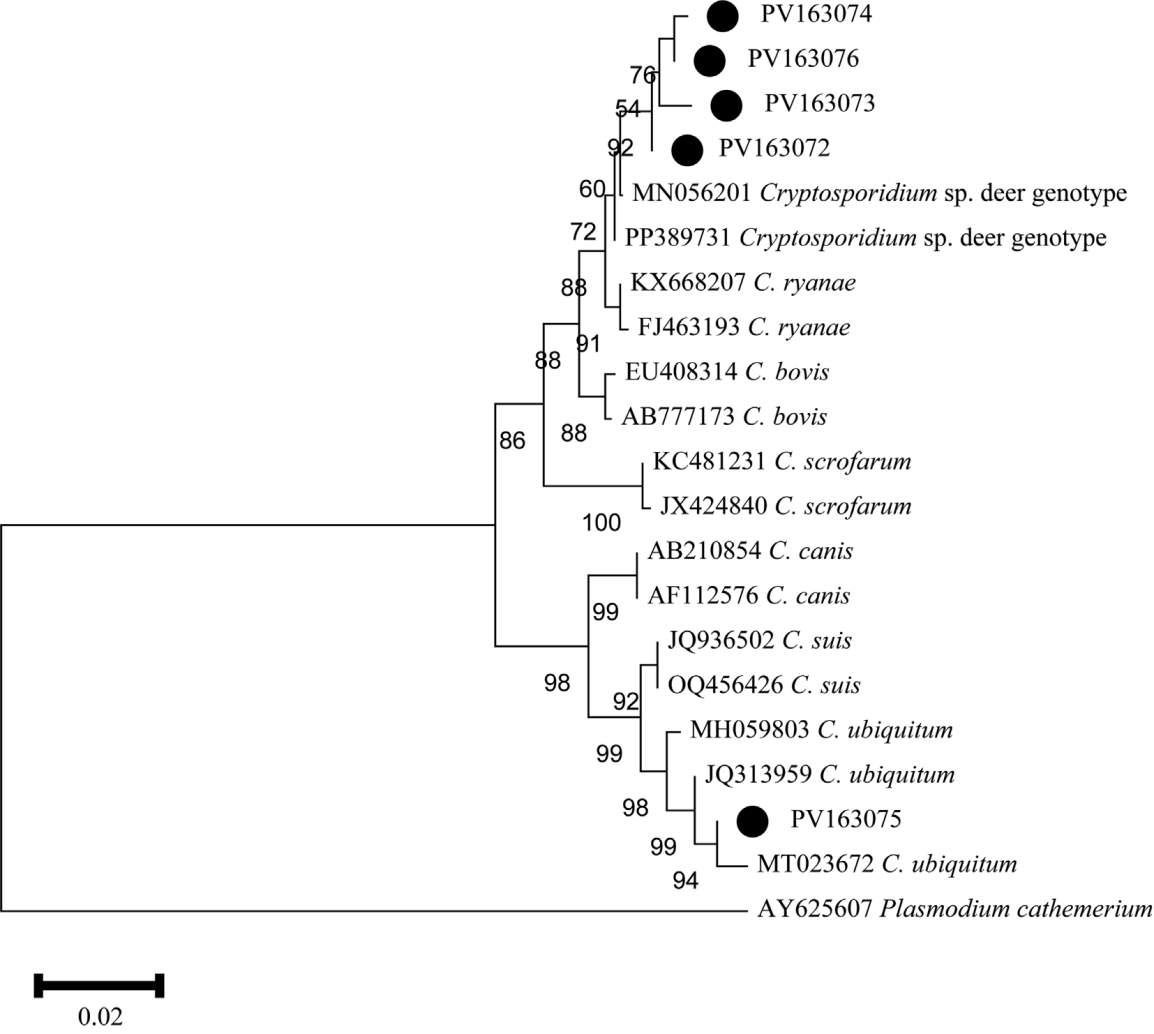
Phylogenetic relationships between the sequences in this study and the reference sequences of *Cryptosporidium* in GenBank and an outgroup, using a neighbor-joining (NJ) method. The genetic distance was calculated based on the Kimura 2-parameter model. Representative nucleotide sequences identified in this study are marked by black dots. The numbers on the branches represent the bootstrap percentage values for 1,000 replicates and are shown in the tree with values greater than 50%.

In this study, after 99% clustering of 69 *Cryptosporidium* sp. sequences, five representative sequences were obtained. These five representative sequences together with 16 *Cryptosporidium* sp. sequences from GenBank and one exogenous sequence were used to construct a phylogenetic tree. The results showed that PV163072, PV163073, PV163074, and PV163076 clustered in the same branch with the reference sequence MN056201. However, there were subtle differences between these representative sequences and the reference sequence MN056201. Specifically, PV163072 has a single nucleotide polymorphism (SNP), PV163073 has two nucleotide deletions, PV163074 had one SNP, one nucleotide insertion, and three nucleotide deletions, and PV163076 had one nucleotide deletion and one insertion. The representative sequence PV163075 and the reference sequence JQ313959 clustered in the same branch and had 100% similarity (Fig. 2).

## Discussion

In this study, the overall infection rate of *Cryptosporidium* sp. in sika deer was 14.81%, which was higher than the global infection rate of deer. For example, the infection rate was 7.5% in sika deer in Japan, 1.5% in red deer in Spain, and 1.42% in Cervidae in Brazil [10, 31, 36]. The infection rate in this study was also higher compared with that in other regions of China, such as the alpine musk deer in Gansu Province (3.9%), Père David’s deer in Jiangsu Province (1.46%), and sika deer in Inner Mongolia, Jilin, Heilongjiang, and Liaoning provinces (13.57%) as a whole [18, 26, 38]. The different infection rates may be due to differences in regional climate, species susceptibility to disease, and sampling time. In addition, in this study, the infection rate of each region ranged from 10.71% to 23.60%, with the highest infection rate in Heilongjiang Province (23.60%), followed by Shandong Province (18.00%), and the lowest infection rate in Jilin Province (10.71%). The sample size may be a factor affecting the difference in infection rates. It is worth noting that the infection rate of young animals (28.21%) in this study was significantly higher than that of adult animals (10.32%), a result consistent with the study of Zhao *et al.*, which may be related to underdeveloped immune function [48].

Phylogenetic analysis revealed close phylogenetic relationships among *C.* deer genotype, *C. ryanae*, *C. bovis*, *C. scrofarum*, *C. canis*, *C. suis*, and *C. ubiquitum*. Based on epidemiological data, *C.* deer genotype has only been reported in Cervidae, such as German red deer, Chinese sika deer, and American white-tailed deer, highlighting its host specificity for Cervidae [19, 34, 40]. In contrast, *C. ryanae*, *C. bovis*, *C. scrofarum*, *C. canis*, and *C. suis* are found globally in a wide range of mammals, such as Turkish buffalo, Ethiopian calf, Japanese sika deer, Chinese pig, Latvian cattle, Korean dog, *etc.* [3, 11, 20, 33, 46]. Notably, *C. ubiquitum* was first identified in white-tailed deer in New York, USA, where it was named *C.* cervine genotype, and subsequently in goats in Hainan and wild rodents on the Qinghai-Tibet Plateau [30, 39, 47]. Additionally, Ong *et al.* first detected the presence of *C. ubiquitum* in humans in 2002. Since then, reports of human infections in this species have gradually increased, with case reports from Sweden, New York, and Spain, *etc.* [1, 4, 9, 29]. These results indicate that *C. ubiquitum* is transmissible across hosts and is zoonotic.

In this study, *C.* deer genotype (98.55%, 68/69) was the dominant species in sika deer, with one case (1.45%) of *C. ubiquitum*. This finding is consistent with the findings of Tao *et al.* [38]. Meanwhile, subtype analysis revealed that the *C. ubiquitum-*positive sample in this study belonged to the XIIa subtype, which is consistent with previous findings in sika deer from northeastern China [48]. This subtype has also been detected in Tibetan sheep from Gansu, camels from Xinjiang, chinchillas from Guangdong, and goats from Inner Mongolia [5, 8, 24, 42]. Globally, it has been reported in sheep from Spain and Australia, as well as goats from Algeria [2, 12, 45]. Notably, this subtype has also been identified in humans, including cases from Ethiopia and the United Kingdom [6, 22]. These findings suggest that the XIIa subtype has cross-species transmission potential and zoonotic capability, indicating that sika deer may serve as a potential reservoir for human *Cryptosporidium* infection.

However, this study still has some limitations, and the sample size in individual areas is small, which may affect an accurate representation of prevalence. Future studies should increase the sample size and use year-round surveillance to fully describe the prevalence of *Cryptosporidium* in sika deer. Notably, previous studies have shown that *C. ubiquitum* can be detected in surface water sources, and in particular, there were reports of *C. ubiquitum* contaminating water sources in the present study area [15, 27]. Therefore, future studies should focus on investigating water sources around sika deer farms to prevent waterborne outbreaks of the disease. In addition, testing of breeding personnel is particularly important to prevent the spread of pathogens to other populations or human communities.

## Conclusion

In conclusion, the results of this study indicate that the overall prevalence of *Cryptosporidium* in sika deer from northern China is 14.81%, suggesting that this parasite is widespread in the local sika deer population. The *C.* deer genotype was identified as the dominant species in sika deer, and the zoonotic *C. ubiquitum* subtype (XIIa) was also identified, suggesting that sika deer may play a role in the transmission of *Cryptosporidium* sp. infection in humans. This study highlights the importance of environmental investigation and personnel screening to provide a basis for risk aversion for public health safety.
